# Observed and Forecasted Antibiotic Consumption by ATC/DDD Metrics: A Real-World Modeling Study with Implications for Antimicrobial Stewardship

**DOI:** 10.3390/antibiotics15080805

**Published:** 2026-08-18

**Authors:** Plamen Bekyarov, Momchil Lambev, Silviya Mihaylova

**Affiliations:** Medical College, Medical University of Varna, 9000 Varna, Bulgaria; momchil.lambev@mu-varna.bg (M.L.); silviya.mihaylova@mu-varna.bg (S.M.)

**Keywords:** antibiotic consumption, predictive modeling, antimicrobial stewardship, hospital pharmacy

## Abstract

**Background/Objectives:** Monitoring antibiotic consumption is a central component of antimicrobial stewardship because it provides a standardized way to identify changing prescribing patterns, detect potentially excessive use, and support targeted interventions. The WHO ATC/DDD framework is widely used for hospital-based utilization studies and enables comparison across wards and time periods. **Methods:** This single-center observational study used antibiotic data obtained from the hospital pharmacy to compare predicted and observed consumption for 2024 and 2025. Antibiotic use was expressed as DDD per 100 bed-days and analyzed for penicillins, cephalosporins, fluoroquinolones, tetracyclines, nitroimidazole derivatives, lincosamides, and aminoglycosides. The aim of this study was to quantify antibiotic consumption using ATC/DDD methodology, evaluate how closely modeled predictions matched observed values in 2024 and 2025, and identify the antibiotic classes and wards most relevant for stewardship action. **Results:** Cephalosporins showed the closest agreement between forecasted and observed consumption, with absolute percentage errors of 2.16% in 2024 and 4.61% in 2025 and a two-year mean absolute percentage error of 3.39%. Consumption of fluoroquinolones exceeded predictions, particularly in gynecology, while use of nitroimidazole derivatives was lower than expected, especially in 2025. Use of tetracyclines was absent in both years, and aminoglycosides and lincosamides contributed only minimally to total consumption. Ward-level analysis showed that intensive care accounted for the highest cephalosporin exposure, whereas the largest deviations from forecast were observed in gynecology and high-risk pregnancy wards. **Conclusions:** Predictive modeling captured antibiotic use reasonably well for some antibiotic classes but showed lower accuracy for others. Forecast accuracy varied across antibiotic groups, indicating that the model performed differently depending on the observed consumption pattern. However, the underlying reasons for these differences cannot be determined from the present aggregated consumption analysis.

## 1. Introduction

Antimicrobial resistance remains one of the most important threats to modern health care, and inappropriate antibiotic use in hospitals is a major driver of this problem. Surveillance of antibiotic consumption is therefore not merely descriptive; it is an operational stewardship tool that helps institutions understand how antibiotics are used, where use is concentrated, and which drug classes are most likely to generate pressure on healthcare systems and the environment [1,2,3,4,5].

In 2017, the World Health Organization (WHO) Expert Committee on the Selection and Use of Essential Medicines developed the Access, Watch, and Reserve (AWaRe) classification of antibiotics to support antimicrobial stewardship (AMS) efforts at the local, national, and global levels. Antibiotics are categorized into three groups (Access, Watch, and Reserve) based on the impact of individual agents and antibiotic classes on the development of antimicrobial resistance, thereby emphasizing the importance of their appropriate use. The classification is updated every two years, with the most recent version published in 2025 [6]. The AWaRe classification is used as a tool for monitoring antibiotic consumption, establishing targets, and assessing the impact of AMS policies aimed at optimizing therapeutic regimens and limiting the emergence and spread of antimicrobial resistance.

At the European level, the European Center for Disease Prevention and Control (ECDC) has also published a set of targets to be achieved by 2030. These recommended targets provide an effective framework for monitoring progress and achieving measurable outcomes in the prevention and reduction of antimicrobial resistance. They are specific and quantifiable and have been established both for the European Union as a whole and for each individual Member State. Key targets include a 20% reduction in total antibiotic consumption in humans and ensuring that at least 65% of total human antibiotic consumption comprises agents from the WHO AWaRe “Access” group [7].

Among available drug-utilization methods, the WHO Anatomical Therapeutic Chemical Classification/Defined Daily Dose system is the most widely used standardized framework for comparing antibiotic use across wards, hospitals, and time periods [8,9,10]. Expressing antibiotic consumption as DDD per 100 bed-days normalizes use according to hospital activity and facilitates descriptive comparisons across wards and time periods, including periods with variations in admissions or length of stay. However, this metric does not account for differences in case mix, patient severity, prescribing indications, individualized dosing, or treatment duration; therefore, such comparisons should be interpreted within the relevant clinical context. The ATC/DDD methodology has been applied in a wide range of clinical settings, including internal medicine, surgery, intensive care, and obstetric and gynecological care [2,11].

The rational use of antibiotics in hospital practice is essential for limiting antimicrobial resistance and optimizing therapeutic outcomes. In response to the global threat posed by antibiotic resistance, AMS has become established as a central component of hospital policy. AMS represents a strategic approach to the appropriate use of antimicrobial agents, aimed at preserving their effectiveness and limiting the emergence and spread of antimicrobial resistance. In hospitals and other healthcare facilities, AMS is implemented through structured programs coordinated by multidisciplinary specialist teams that seek to optimize antimicrobial prescribing and use. These programs typically include the development of evidence-based treatment guidelines, monitoring of antimicrobial consumption, and the implementation of educational initiatives for healthcare professionals [12]. The implementation of AMS at the national, regional, community, or institutional level requires a range of actions and methods, including regulatory instruments, such as policies and guidelines; legislative measures, such as binding regulations; and locally adapted strategies tailored to the specific healthcare context. Within healthcare institutions, AMS plays a key role in reducing inappropriate antibiotic use and minimizing associated risks, including infections caused by multidrug-resistant pathogens [13].

Invasive obstetric and gynecological procedures are associated with an increased risk of surgical site infections, which may substantially affect patient health and the course of postoperative recovery. Antibiotic prophylaxis has been shown to reduce the incidence of postoperative infections, particularly in procedures associated with an increased likelihood of bacterial contamination, such as cesarean delivery, hysterectomy, and other invasive obstetric and gynecological interventions. Examining antibiotic prophylaxis in the context of obstetric and gynecological procedures is particularly relevant to the objectives of the present study, as the analysis of hospital antibiotic use will enable an assessment of compliance with good clinical practice and identify opportunities for optimizing institutional antibiotic policy.

Antibiotic prophylaxis is generally administered as a single dose in accordance with the relevant pharmacotherapeutic guidelines. A study published as early as 1992 demonstrated that there is a limited time window within which effective antimicrobial prophylaxis can be administered [14]. To ensure efficacy, adequate antibiotic concentrations must be present in the tissues at the time of surgical incision. In obstetric and gynecological practice, the administration of additional antibiotic doses beyond the initial dose given at the induction of anesthesia is rarely required. Most studies comparing single-dose and multiple-dose prophylactic strategies have not demonstrated a significant benefit of repeated administration, although these investigations were not exclusively limited to obstetric and gynecological procedures. According to Clifford and Daley, intraoperative redosing should be considered only when the duration of the procedure exceeds the elimination half-life of the administered antimicrobial agent [15].

Descriptive surveillance alone, however, does not answer a key practical question: what should antibiotic use look like if prescribing patterns remain stable? That is where predictive modeling adds value. In hospital-level antibiotic studies, linear trend analysis, time-series methods, and forecast intervals have been used to estimate expected use and compare it with observed consumption during later periods. Such comparisons are especially informative when an antibiotic class is known to be sensitive to institutional policy, case mix, or local prescribing habits, as is the case for fluoroquinolones and metronidazole [16,17,18,19,20,21].

## 2. Results

Comparison of forecasted and observed antibiotic consumption across the seven analyzed classes reveals three distinct behavioral patterns that persisted, evolved, or reversed between 2024 and 2025. To quantitatively assess the agreement between forecasted and observed consumption across these antibiotic groups, APE values for 2024 and 2025 and the corresponding two-year MAPE are presented in Table 1.

**Figure 1:**
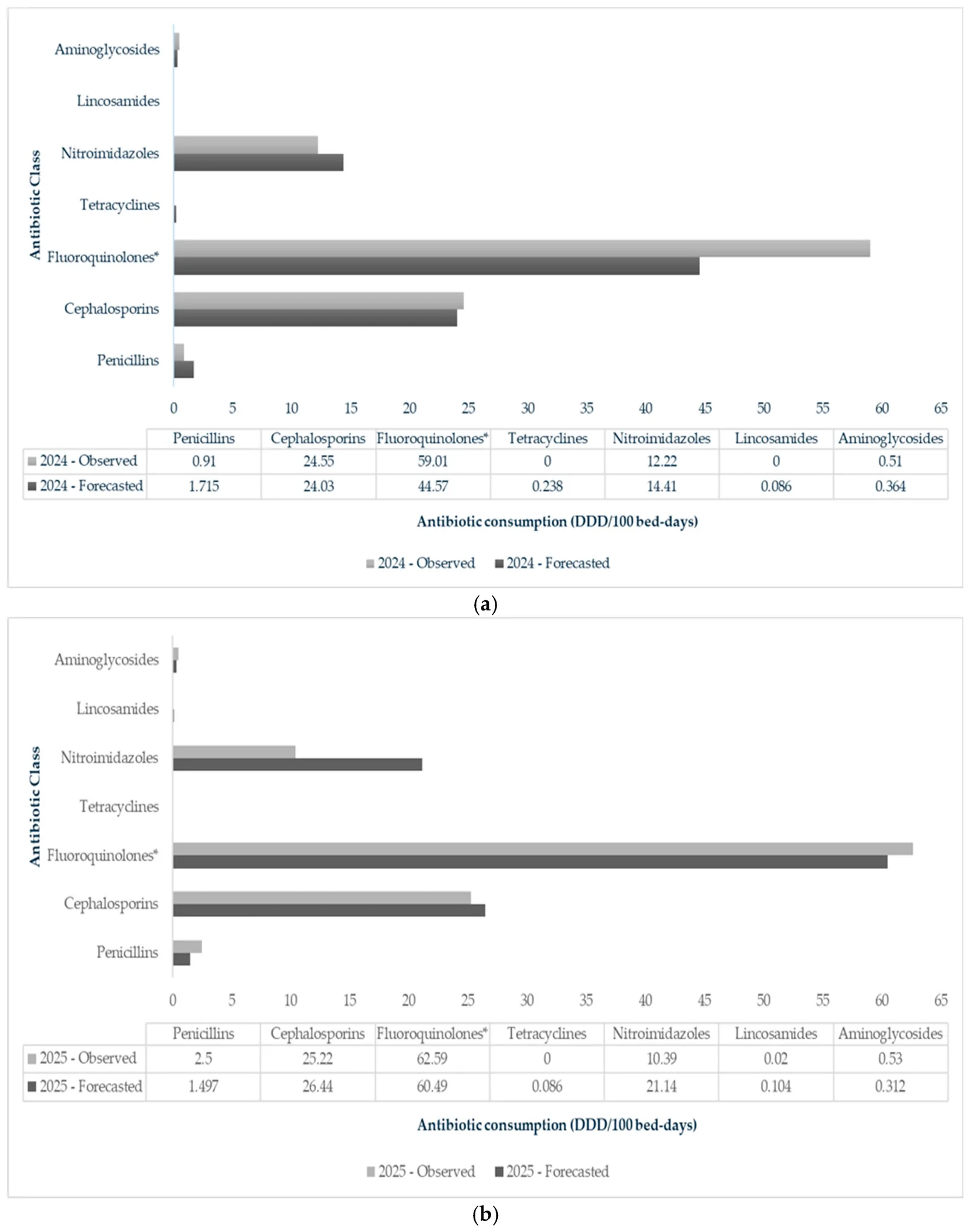
(**a**) Forecast and Observed Hospital Antibiotic Consumption by Antibiotic Class in 2024 (DDD per 100 Bed-Days). (**b**) Forecast and Observed Hospital Antibiotic Consumption by Antibiotic Class in 2025 (DDD per 100 Bed-Days). * Fluoroquinolone consumption was concentrated in the Gynecology Ward, with minimal use in the remaining wards.

In 2024 (Figure 1a), fluoroquinolones exhibited the most pronounced and clinically significant deviation from the model, with observed consumption (59.01 DDD/100 bed-days) substantially exceeding the forecasted value (44.57 DDD/100 bed-days), corresponding to an absolute difference of 14.44 DDD/100 bed-days and a relative deviation of approximately 32%.

This magnitude of higher-than-forecast consumption was unmatched by any other antibiotic class in the dataset and immediately identified fluoroquinolones as the primary target for stewardship intervention. Use of cephalosporins demonstrated near-perfect concordance between forecasted (24.03 DDD/100 bed-days) and observed (24.55 DDD/100 bed-days) values, reflecting a stable, protocol-driven prescribing pattern consistent with institutionalized empirical practice. Use of nitroimidazole derivatives in 2024 was modestly below prediction (12.22 DDD/100 bed-days observed versus 14.41 DDD/100 bed-days forecasted), while penicillins showed the opposite pattern, with observed use (0.91 DDD/100 bed-days) falling below the forecasted level (1.715 DDD/100 bed-days). Tetracyclines, lincosamides, and aminoglycosides remained at negligible absolute levels throughout the year, contributing no clinically meaningful deviation.

The 2025 data indicate that the pattern was neither uniform nor stable across all classes (Figure 1b). Fluoroquinolone use continued to exceed the forecast with approximately 3.5% relative deviation between observed (62.59 DDD/100 bed-days) and forecasted (60.49 DDD/100 bed-days). This apparent convergence should not be interpreted as evidence of improved prescribing discipline; rather, it reflects the forecasting model adjusting to an already elevated baseline established in the preceding year, while absolute consumption remained persistently high throughout the study period. Cephalosporins retained their characteristic stability, with observed use (25.22 DDD/100 bed-days) again closely tracking the forecast (26.44 DDD/100 bed-days). Nitroimidazole derivatives displayed the most substantial shift observed in the entire dataset: consumption fell to nearly half of the predicted value (10.39 DDD/100 bed-days observed versus 21.14 DDD/100 bed-days forecasted), suggesting either a marked reduction in anaerobic coverage requirements or a shift toward alternative therapeutic regimens. Penicillins reversed their 2024 trend entirely, with observed use (2.50 DDD/100 bed-days) now exceeding the forecast (1.497 DDD/100 bed-days). Tetracyclines and lincosamides remained essentially absent from clinical use, while aminoglycosides continued to show slightly elevated consumption relative to prediction in both years.

Across the two validation years, cephalosporins had the lowest MAPE (3.39%), with APEs of 2.16% in 2024 and 4.61% in 2025. This descriptive error comparison, rather than visual inspection alone, supports their characterization as the group with the closest and most consistent agreement between forecasted and observed consumption (Table 1).

When considered together, the two-year trajectory delineates three functionally distinct categories of antibiotic behavior with direct implications for AMS planning. Cephalosporins represent a stable antibiotic class in which forecasting accurately reflects institutional prescribing practice, likely attributable to well-established empirical protocols in high-intensity settings such as the intensive care unit (ICU). Use of fluoroquinolones is characterized by sustained higher-than-forecast consumption across both years; the apparent narrowing of relative deviation in 2025 is a statistical artifact of model recalibration to an elevated baseline rather than a genuine reduction in prescribing intensity, and absolute consumption levels exceeding 59 DDD/100 bed-days throughout the study period identify this class as the single most actionable target for a formal stewardship intervention, particularly given its documented concentration within the gynecology ward (GW). Nitroimidazole derivatives and penicillins, finally, exhibit an unstable and non-directional pattern, with each class reversing its deviation direction between the two years; this volatility suggests that consumption of these agents is governed more by shifting clinical practice, seasonal case mix, or evolving local guidelines than by a consistent temporal trend, limiting the reliability of forecast-based surveillance for these two classes and warranting closer indication-level investigation before any stewardship action is proposed.

Ward-level analysis of cephalosporin consumption reveals a considerably more heterogeneous picture than the hospital-wide aggregate suggests, with distinct patterns emerging across the four monitored wards in both study years.

**Figure 2:**
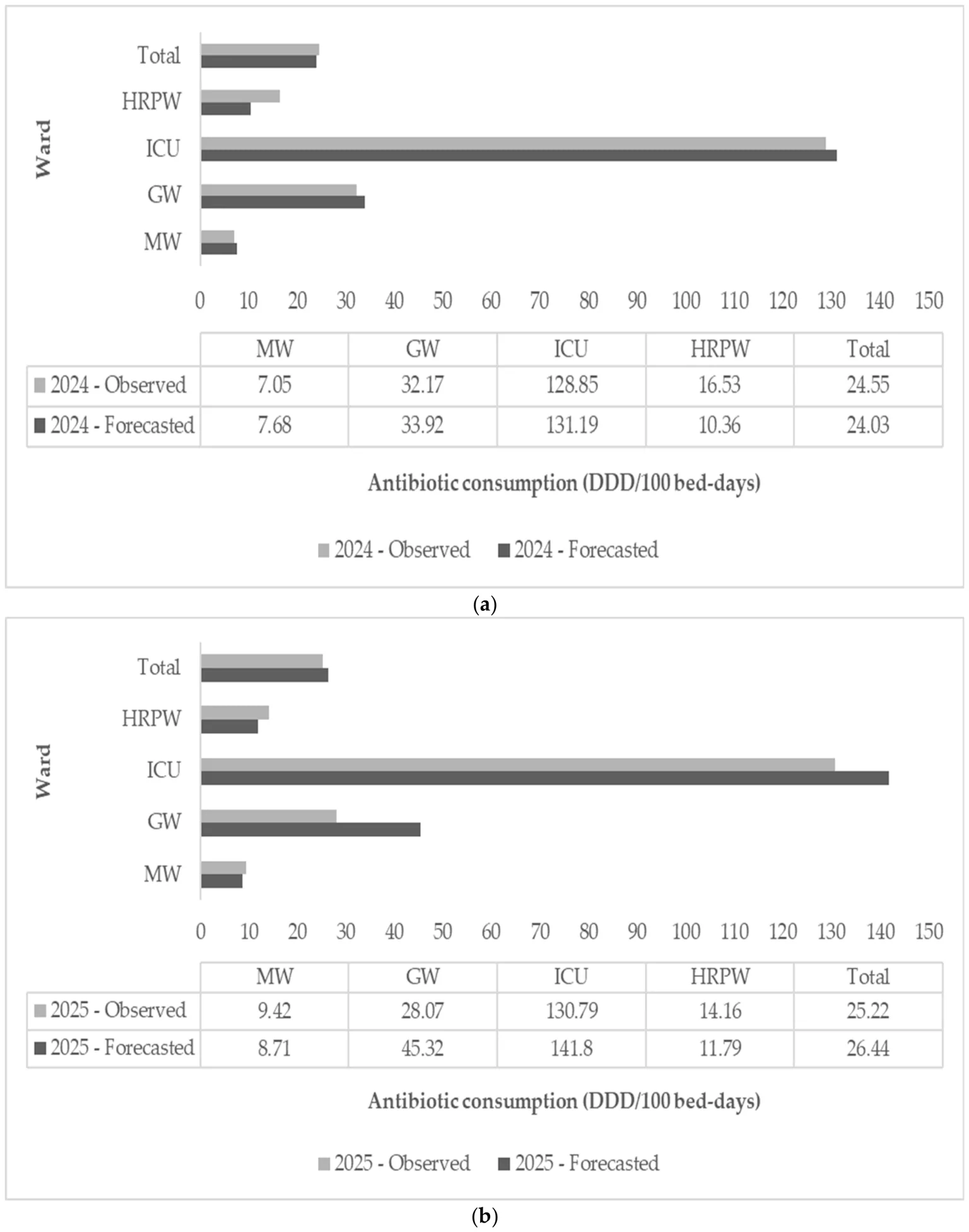
(**a**) Ward-Level Predicted and Observed Cephalosporin Consumption in 2024 (DDD per 100 Bed-Days). (**b**) Ward-Level Predicted and Observed Cephalosporin Consumption in 2025 (DDD per 100 Bed-Days).

In 2024 (Figure 2a), ICU accounted for by far the highest overall cephalosporin exposure, with observed consumption (128.85 DDD/100 bed-days) closely tracking the forecasted value (131.19 DDD/100 bed-days), confirming a stable prescribing culture in this high-acuity setting. The GW showed similarly close alignment between observed (32.17 DDD/100 bed-days) and forecasted (33.92 DDD/100 bed-days) values, while the maternity ward (MW) exhibited comparably modest and well-matched consumption (7.05 DDD/100 bed-days observed versus 7.68 DDD/100 bed-days forecasted). Notably, the high-risk pregnancy ward (HRPW) displayed positive deviation in 2024, with observed use (16.53 DDD/100 bed-days) substantially exceeding the forecast (10.36 DDD/100 bed-days), foreshadowing a pattern of local higher-than-forecast consumption that would persist into the following year.

The 2025 data (Figure 2b) indicate that this ward-level heterogeneity became more pronounced. Obviously, the ICU demonstrates the highest cephalosporin exposure and retained reasonably close concordance with the forecast (130.79 DDD/100 bed-days observed versus 141.80 DDD/100 bed-days forecasted), reaffirming the overall stability of prescribing practice in this ward despite its high consumption intensity. In the GW, observed consumption of cephalosporins in 2025 was 28.07 DDD/100 bed-days, which was 17.25 DDD/100 bed-days below the forecasted value of 45.32 DDD/100 bed-days, corresponding to a relative deviation of −38.1%. This represented the largest ward-level deviation observed for cephalosporins in either study year.

The MW showed a modest increase in observed use relative to forecast (9.42 DDD/100 bed-days versus 8.71 DDD/100 bed-days), while HRPW again exceeded its forecasted value (14.16 DDD/100 bed-days observed versus 11.79 DDD/100 bed-days forecasted), consistent with the positive forecast deviation already identified in 2024.

The two-year ward-level trajectory demonstrates that hospital-wide stability in cephalosporin consumption is an aggregate phenomenon that obscures clinically meaningful heterogeneity at the ward level, which must be considered when implementing an AMS program to reduce AMR. The ICU had the highest observed consumption of cephalosporins among the four analyzed wards in both 2024 and 2025. The higher consumption of cephalosporins observed in the ICU may reflect the clinical characteristics of the patients treated in this ward and may also be partly related to local cefazolin-based antibiotic prophylaxis practices. However, the relative contribution of these factors was not assessed separately in the present study. The HRPW displayed persistent and directionally consistent higher-than-forecast consumption in both 2024 and 2025, identifying it as a candidate for targeted stewardship review independent of the reassuring hospital-wide totals. The GW presents the most valuable finding of the ward-level analysis: a reversal from close forecast alignment in 2024 to substantial underuse relative to prediction in 2025, a pattern that merits further investigation to determine whether it reflects a deliberate stewardship-driven reduction in antibiotic use, a shift in case mix, or changes in the distribution of prescribing of cephalosporins across individual antibiotic classes within the ward. The MW remained the most stable and lowest-intensity setting throughout the study period, with only minor deviations in either direction. These findings collectively underscore the importance of ward-stratified surveillance of antibiotic consumption as a necessary complement to hospital-wide ATC/DDD monitoring, since institutional-level stability can mask ward-specific prescribing patterns that carry direct implications for the design and prioritization of AMS interventions.

**Figure 3:**
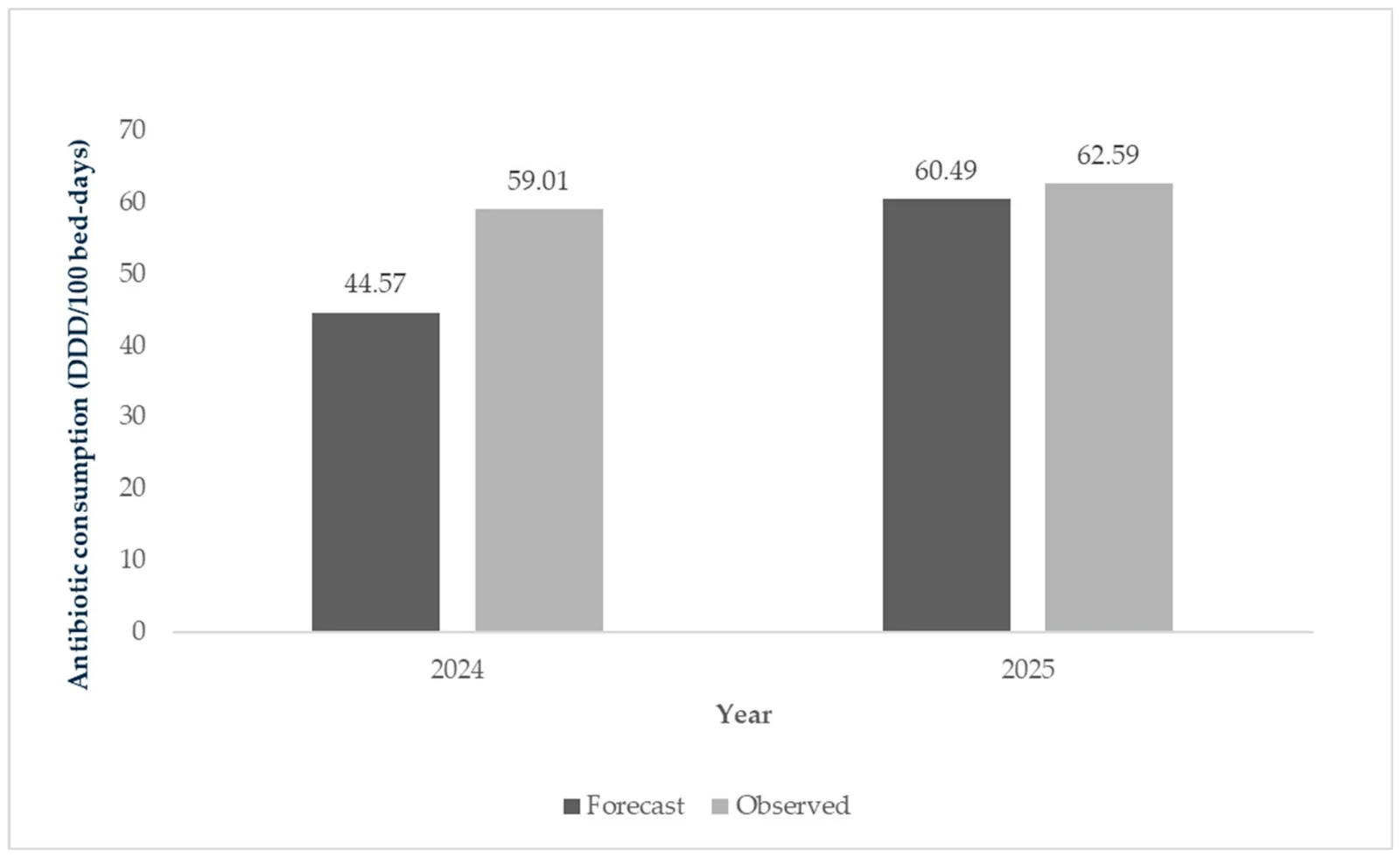
Forecast and Observed Fluoroquinolone Consumption in the GW in 2024 and 2025 (DDD per 100 Bed-Days).

Figure 3 illustrates the discrepancy between forecasted and observed fluoroquinolone consumption in the GW across the two study years. Observed fluoroquinolone consumption exceeded the forecast in both 2024 (+32.4%) and 2025 (+3.5%), with the magnitude of the deviation narrowing considerably between the two years. In 2024, observed fluoroquinolone consumption in the GW was 59.01 DDD/100 bed-days, exceeding the forecasted value of 44.57 DDD/100 bed-days by 14.44 DDD/100 bed-days, corresponding to a relative deviation of +32.4%, representing the single largest deviation recorded in this study. In 2025, observed consumption (62.59 DDD/100 bed-days) was close to the forecasted value (60.49 DDD/100 bed-days), indicating substantially improved forecast agreement compared with 2024. Although absolute consumption remained high, these findings should not be interpreted as evidence of persistent excess consumption.

This pattern suggests that, while the forecasting model continued to underestimate the true magnitude of fluoroquinolone use in this ward, the observed deviation may reflect ward-specific prescribing factors rather than a stable temporal trend alone.

## 3. Discussion

This study demonstrates that combining routine ATC/DDD surveillance with predictive modeling can reveal both stable and problematic antibiotic-use patterns that would remain hidden in purely descriptive reporting [22]. The close agreement between forecasted and observed cephalosporin consumption, particularly in the ICU, suggests a protocol-driven and relatively predictable prescribing culture for this class, which is consistent with previous hospital-based DDD studies showing that beta-lactam use tends to follow stable institutional patterns once empirical protocols are established [23].

The positive fluoroquinolone forecast deviation identified in the GW may be used to prioritize a prescription-level review of indication, dosing, duration, and guideline concordance. This mirrors international experience: fluoroquinolones have repeatedly been identified as the antibiotic class most amenable to targeted stewardship interventions, because their high levels of use are strongly associated with resistance selection in Gram-negative pathogens [24]. Pharmacist-led stewardship programs specifically targeting fluoroquinolone restriction have achieved reductions in empirical fluoroquinolone prescribing of up to 30%, together with measurable improvements in antimicrobial susceptibility and reduced attributable mortality [25]. The dataset did not include prescription-level indications; therefore, the reasons for the higher-than-forecast fluoroquinolone consumption in the GW cannot be determined from the available aggregated data. Possible explanations, including prophylactic and empirical use, should be regarded as hypotheses for future prescription-level investigation rather than as established findings.

A possible explanation for the disproportionately high consumption of cephalosporins observed in this study relates to their established role as the recommended agents for antibiotic prophylaxis in gynecological surgery, including abdominal and vaginal hysterectomy and cesarean section, in accordance with the guidelines issued by the Bulgarian Association of Microbiologists [26] for antibiotic prophylaxis in obstetric-gynecological interventions. On the other hand, antibiotic prophylaxis in obstetric-gynecological interventions is a key component in the prevention of healthcare-associated infections. Evaluating its implementation in the hospital setting makes it possible to assess compliance with clinical guidelines and highlights the potential for improving antimicrobial policy.

Observed consumption of nitroimidazole derivatives in 2025 was 10.39 DDD/100 bed-days, which was 10.75 DDD/100 bed-days below the forecasted value of 21.14 DDD/100 bed-days, corresponding to a relative deviation of −50.9%. The reasons for this difference cannot be determined from the available aggregated data. Potential explanations, including changes in indications, surgical practice, local guidance, or substitution by other antimicrobial regimens, require prospective investigation. This finding is consistent with reports that antibiotic classes historically used for prophylaxis are particularly sensitive to changes in surgical practice and local guideline updates over short periods [8,24].

From a methodological standpoint, our results align with published evidence that dynamic and time-series forecasting models perform well for antibiotic classes with stable, protocol-driven use but lose accuracy for classes influenced by ward-specific clinical practice or evolving prescriber behavior [27]. Compared descriptively with previously reported prediction-error ranges of approximately 8–34% in hospital-based forecasting studies [24], forecast errors for cephalosporins in the present study were lower (2.2% in 2024 and 4.6% in 2025). The 2024 fluoroquinolone error was within this range (32.4%), whereas the 2025 error was lower (3.5%). In contrast, the 2025 error for nitroimidazole derivatives was higher (50.9%). This comparison should be interpreted cautiously because of differences in model structure, data frequency, outcome definition, and clinical setting across studies. This supports our interpretation that the value of predictive modeling in this context lies primarily in flagging classes and wards that deviate meaningfully from expected use, rather than in generating exact utilization forecasts.

These findings have direct implications for AMS implementation at the study hospital. The Core Elements of Hospital Antibiotic Stewardship Programs emphasize leadership commitment, pharmacy expertise, tracking and reporting of antibiotic use, and action on identified problems as central pillars of an effective program [28]. Our data provide an evidence base for at least three concrete stewardship actions: (1) establishing a gynecology-specific review of fluoroquinolones and pre-authorization requirement, given that this ward alone accounted for the largest deviation from forecast; (2) using the stability of cephalosporins as a benchmark against which future interventions in other classes can be measured; and (3) incorporating routine forecast-versus-observed comparison into the hospital’s stewardship dashboard, allowing the multidisciplinary stewardship team to prioritize review of wards and classes showing the greatest divergence, rather than reviewing all prescriptions uniformly. This targeted approach is consistent with evidence that culture-guided, deescalation-focused stewardship interventions can reduce DDD/100 bed-days, cost, length of stay, and resistance rates within a relatively short implementation period [29].

A key interpretive limitation of this study is the absence of indication-level, microbiological, case-mix, severity, dose, and duration data, which limits the assessment of the clinical appropriateness of observed antibiotic consumption. Forecast deviations should therefore be interpreted as exploratory signals that may help identify antibiotic groups or areas warranting further review, rather than as standalone evidence of overuse or definitive stewardship priorities. The single-center design limits generalizability to hospitals with different case mixes or prescribing cultures [22]. The absence of microbiological, indication-level, and patient-severity data prevents distinguishing appropriate from inappropriate use within each antibiotic class, and the DDD methodology itself does not capture individualized dosing, which is particularly relevant in obstetric populations where weight-based or renal-adjusted dosing is common [8]. Future work should integrate ward-level clinical indication data and microbiological susceptibility trends to allow the forecasting approach to be linked directly to resistance outcomes.

## 4. Materials and Methods

A single-center observational study was conducted, combining retrospective analysis of observed antibiotic use with model-based prediction of utilization for 2024 and 2025. The study was designed to compare forecasted values with actual consumption in routine clinical practice and to assess the practical performance of the model rather than to test causal intervention. The study was conducted in a specialized hospital for active treatment in obstetrics and gynecology serving Northeastern Bulgaria and providing obstetric, gynecological, neonatal, and intensive care services. The hospital has a total capacity of 140 beds. The total number of occupied patient bed-days was 12,598 in 2024 and 12,443 in 2025. No major changes in institutional antibiotic policy or antibiotic prophylaxis protocols were introduced during the study period.

### 4.1. Data Source

Data were obtained from the hospital pharmacy. The dataset included annual antibiotic consumption values by major class and ward-level values for selected antibiotic groups, particularly cephalosporins and fluoroquinolones, allowing both aggregate and ward-level analyses. This approach is consistent with standard ATC/DDD-based hospital utilization studies, in which antibiotic use is commonly derived from pharmacy or prescribing databases and then normalized to hospital activity measures such as bed-days.

Permission to process and analyze the data was obtained from the hospital administration. The study was approved by the Ethics Committee of the hospital (Protocol No. 26, 22 January 2026). Only aggregated, non-identifiable hospital pharmacy-consumption and hospital-activity data were analyzed, and no personal patient identifiers or individual patient-level records were included in the analytical dataset.

For the present analysis, pharmacy-based data were preferred because they provide a systematic and routinely collected source for hospital drug-use surveillance, while ward-level stratification enabled identification of ward-specific patterns that would not be visible in hospital-wide totals alone. Such a structure is particularly useful in studies of broad-spectrum antibiotics, because use may differ substantially across wards depending on case mix, prophylactic protocols, and local empirical prescribing habits.

### 4.2. Antibiotic Classes Analyzed

The following antibiotic classes were included in the analysis: tetracyclines (J01A), penicillins (J01C), cephalosporins (J01D), lincosamides (J01FF), aminoglycosides (J01G), fluoroquinolones (J01MA), and imidazole derivatives (J01XD). These classes were selected because they accounted for the main observed and modeled antibiotic-use patterns in the hospital dataset and because several of them are clinically important stewardship targets.

A total of 12 antibiotic agents or combinations were included. Nine agents (75.0%) belonged to the WHO AWaRe Access group, whereas three agents (25.0%)-cefuroxime, ceftriaxone, and ciprofloxacin belonged to the Watch group. No antibiotics from the WHO AWaRe Reserve group were used in the hospital during the study period. The selected agents, their complete ATC codes, WHO Defined Daily Dose values, routes of administration used in the hospital (oral or parenteral), and AWaRe categories are presented in Table 2.

### 4.3. Outcome Measure

The primary outcome was total antibiotic consumption expressed as DDD per 100 occupied patient bed-days, following the WHO ATC/DDD methodology. For each antibiotic, the number of consumed DDDs was calculated using the following formula:

$$Consumed\;DDDs=\frac{number\;of\;used\;dosage\;forms\;(vials,\;ampoules,\;tablets)\;\times \;amount\;of\;active\;substance\;per\;unit\;(g)\;}{defined\;DDD\;WHO\;(g)}$$

Total antibiotic consumption was subsequently calculated as follows:

$$Antibiotic\;consumption\;=\;\frac{consumed\;DDDs}{total\;occupied\;patient\;beddays}\;\times \;100$$

The WHO-defined DDD corresponding to the route of administration used in the hospital was applied. Occupied patient bed-days, rather than the number of available beds, were used as the denominator. The total numbers of occupied patient bed-days were 12,598 in 2024 and 12,443 in 2025. This measure standardizes antibiotic consumption according to hospital activity and enables comparisons across study periods.

### 4.4. Statistical Modeling and Forecasting

Forecasted antibiotic consumption was generated using regression-based curve estimation in IBM SPSS Statistics (version 19). Annual antibiotic consumption data from 2012 to 2023 were used as the model-development period, providing 12 equally spaced observations for each antibiotic series. Time was entered as the independent variable, with t = 1 corresponding to 2012 and t = 12 to 2023. Linear, logarithmic, inverse, quadratic, cubic, power, compound, S-curve, logistic, growth, and exponential functions were evaluated separately for each antibiotic series. Model fit was assessed using the correlation coefficient (R), coefficient of determination (R^2^), adjusted R^2^, standard error of the estimate, and ANOVA. The model providing the best fit and lowest estimation error was selected when statistically significant and subsequently used for forecasting. The selected models, fitted equations, and corresponding model-fit statistics are presented in Table 3.

Forecasts for 2024 and 2025 were obtained by extrapolating the respective fitted equations to t = 13 and t = 14. The original modeling procedure also generated a forecast for 2026 (t = 15), although the present study evaluates forecast performance only for 2024 and 2025, for which observed consumption data were available.

Forecast performance was evaluated by comparing the forecasted values with the subsequently observed consumption values for 2024 and 2025. Percentage deviation was calculated using the forecasted value as the denominator:

$$Percentage\;deviation\;\left(\%\right)\;=\;\frac{Observed\;consumption\;-\;Forecasted\;consumption}{Forecasted\;consumption}\;\times \;100$$

Positive values indicated observed consumption above the forecast, whereas negative values indicated observed consumption below the forecast. Absolute percentage error (APE) was calculated separately for each year, and the mean absolute percentage error (MAPE) across the two years was used as a summary measure of relative forecast accuracy. The forecasting models were used to establish expected consumption based on historical trends and were not intended to determine the clinical appropriateness of antibiotic use.

$$APE\;=\;\frac{\left|observed\;-\;forecast\right|}{forecast}\;\times \;100$$

This descriptive-comparative framework is consistent with previously published hospital-based antibiotic utilization studies that have applied trend analysis and forecast/prediction intervals to benchmark observed consumption against expected values in subsequent periods, thereby supporting the identification of antibiotic classes or wards warranting closer stewardship attention.

## 5. Conclusions

This single-center study shows that combining WHO ATC/DDD surveillance with predictive modeling can identify both stable and higher-than-forecast patterns of antibiotic consumption relevant to AMS. The use of cephalosporins was consistent and well predicted, whereas the consumption of fluoroquinolones in GW substantially and persistently exceeded forecasted levels, supporting its prioritization for prescription-level review. The use of nitroimidazole derivatives fell below prediction, warranting further indication-level investigation, while other classes contributed only marginally to overall consumption.

These results may support the development of targeted AMS activities at the study hospital, with particular attention to the use of fluoroquinolones in gynecology and continued monitoring of the use of cephalosporins in intensive care. Embedding forecast-versus-observed analysis into routine pharmacy surveillance may provide a practical approach for identifying antibiotic classes and wards warranting closer review. Prospective validation incorporating microbiological and clinical-indication data is recommended to strengthen the link between consumption forecasting and resistance-outcome-driven stewardship decisions.

## Figures and Tables

**Table 1:** Descriptive forecast-error comparison across antibiotic groups.

Antibiotic Group	APE 2024 (%)	APE 2025 (%)	Two-Year MAPE (%)
Penicillins	46.94	67.00	56.97
Cephalosporins	2.16	4.61	3.39
Fluoroquinolones	32.40	3.47	17.94
Tetracyclines	100.00	100.00	100.00
Nitroimidazole derivatives	15.20	50.85	33.02
Lincosamides	100.00	80.77	90.38
Aminoglycosides	40.11	69.87	54.99

APE = absolute percentage error; MAPE = mean absolute percentage error; APE was calculated using the forecast as the denominator.

**Table 2:** Selected antibiotic classes/substances for systemic use.

Antibiotic Classes	Antibiotic (INN)	WHO DDD * (g)		AWaRe Group **
		Oral	Parenteral	
J01A Tetracyclines	J01AA02 Doxycycline	0.1	-	Access
J01C Penicillins J01CA Penicillins with extended spectrum	J01CA01 Ampicillin	-	6.0	Access
	J01CA04 Amoxicillin	1.5	-	Access
J01CR Combinations of penicillins, including beta-lactamase inhibitors	J01CR01 Ampicillin/sulbactam	-	6.0	Access
J01DB/DC/DD Cephalosporins	J01DB04 Cefazolin	-	3.0	Access
	J01DC02 Cefuroxime	-	3.0	Watch
	J01DD04 Ceftriaxone	-	2.0	Watch
J01FF Lincosamides	J01FF01 Clindamycin	-	1.8	Access
J01G Aminoglycoside antibacterials	J01GB03 Gentamicin	-	0.24	Access
	J01GB06 Amikacin	-	1.0	Access
J01MA Fluoroquinolones	J01MA02 Ciprofloxacin	1.0	-	Watch
J01XD Imidazole derivatives	J01XD01 Metronidazole	-	1.5	Access

* ATC/DDD Index 2026 [30]. ** AWaRe classification of antibiotics for evaluation and monitoring of use, 2023 [31].

**Table 3:** Regression models used to generate the analyzed antibiotic-consumption forecasts.

Antibiotic Series	Selected Model	Fitted Equation	R^2^	*p*-Value
Penicillins—total	Linear	Y = −0.218t + 4.549	0.749	<0.001
Cephalosporins—total	Quadratic	Y = 0.118t^2^ − 0.781t + 14.245	0.686	0.005
Fluoroquinolones—GW	Cubic	Y = 0.086t^3^ − 1.433t^2^ + 7.567t − 0.567	0.606	0.049
Tetracyclines—total	Cubic	Y = −0.003t^3^ + 0.079t^2^ − 0.644t + 1.850	0.872	0.001
Nitroimidazole derivatives—GW	Cubic	Y = 0.041t^3^ − 0.672t^2^ + 2.443t + 6.143	0.624	0.041
Lincosamides—total	Quadratic	Y = 0.001t^2^ − 0.009t + 0.034	0.762	0.002
Aminoglycosides—total	Exponential	Y = 2.644 × 10^−0.153t^	0.669	0.001

## Data Availability

The data presented in this study are available within the article. No additional datasets were generated.

## Abbreviations

The following abbreviations are used in this manuscript:

WHO ATC/DDD	World Health Organization Anatomical Therapeutic Chemical Classification System/Defined Daily Dose
MW	maternity ward
GW	gynecology ward
ICU	intensive care unit
HRPW	high-risk pregnancy ward
AMS	antimicrobial stewardship
APE	absolute percentage error
MAPE	mean absolute percentage error
